# *Batrachochytrium salamandrivorans*’ Amphibian Host Species and Invasion Range

**DOI:** 10.1007/s10393-022-01620-9

**Published:** 2023-01-07

**Authors:** Federico Castro Monzon, Mark-Oliver Rödel, Florian Ruland, Gabriela Parra-Olea, Jonathan M. Jeschke

**Affiliations:** 1 Institute of Biology, Freie Universität Berlin, Königin-Luise-Str. 1-3, 14195 Berlin, Germany; 2 Leibniz-Institute of Freshwater Ecology and Inland Fisheries, Müggelseedamm 310, 12587 Berlin, Germany; 3 Berlin-Brandenburg Institute of Advanced Biodiversity Research, Altensteinstr. 34, 14195 Berlin, Germany; 4 Department of Zoology, Institute of Biology, Universidad Nacional Autonoma de Mexico, AP 70-153, 04510 Mexico City, Mexico; 5 Museum für Naturkunde–Leibniz Institute for Evolution and Biodiversity Science, Invalidenstr. 43, 10115 Berlin, Germany

**Keywords:** Emerging infectious disease, *Batrachochytrium salamandrivorans*, chytridiomycosis, chytrid, amphibian, systematic review

## Abstract

*Batrachochytrium salamandrivorans (Bsal),* a species related to the destructive pathogen *Batrachochytrium dendrobatidis (Bd),* was found and identified in Europe in 2013. Now, a decade later, a large amount of information is available. This includes data from studies in the field, reports of infection in captive amphibians, laboratory studies testing host susceptibility, and data from prospective studies that test for *Bsal*’s presence in a location. We conducted a systematic review of the published literature and compiled a dataset of *Bsal* tests. We identified 67 species that have been reported positive for *Bsal,* 20 of which have a threatened conservation status. The distribution of species that have been found with infection encompasses 69 countries, highlighting the potential threat that *Bsal* poses. We point out where surveillance to detect *Bsal* have taken place and highlight areas that have not been well monitored. The large number of host species belonging to the families Plethodontidae and Salamandridae suggests a taxonomic pattern of susceptibility. Our results provide insight into the risk posed by *Bsal* and identifies vulnerable species and areas where surveillance is needed to fill existing knowledge gaps.

**Supplementary Information:**

The online version contains supplementary material available at 10.1007/s10393-022-01620-9.

## Introduction

The amphibian chytrid pathogen *Batrachochytrium salamandrivorans* (*Bsal*) presents a risk to amphibian populations across the world. The pathogen has been hypothesized to have originated in East Asia (Martel et al. 2014) and was first identified in 2013 in a region bordering Belgium and the Netherlands (Martel et al. 2013); however, museum records later showed that it was already present in Germany by 2004 (Lötters et al. 2020a). *Bsal* has persisted in the locations where it was first found and has been associated with population collapse of the fire salamander (*Salamandra salamandra*). Since its discovery, the pathogen has also been found in the wild in Germany and in several Asian countries (Nguyen et al. 2017; Laking et al. 2017, Yuan 2017), and there is evidence that it is expanding its range in Europe (Spitzen-van der Sluijs et al. 2016). Recently, it was also reported in Spain by two independent research groups in different regions of the country (Lastra González et al. 2019; Martel et al. 2020).

The pathogen has also been found in captive animals in several countries (Martel et al. 2014; Fitzpatrick et al. 2018; Sabino-Pinto et al. 2018). The presence of *Bsal* in captive animals has been pointed out as a possible source of infection in the wild (Martel et al. 2014). In addition, susceptible species that are distributed outside the current known range of the pathogen have been successfully infected in laboratory exposure experiments, and *Bsal*-related deaths have been observed in a number of them (Martel et al. 2014; Friday et al. 2020).

The potential threat that *Bsal* poses is highlighted by the damage that a related pathogen, *B. dendrobatidis* (*Bd*), has inflicted on amphibian populations. *Bd* has been found all over the world, presumably also spreading from Asia, and has been linked to amphibian declines in many regions (Skerratt et al. 2007; Scheele et al. 2019). *Bd* so far has over 1000 known host species (Castro Monzon et al. 2020) and has been deemed as the disease with the greatest impact on vertebrate biodiversity (e.g., Bellard et al. 2016).

In response to the threat that *Bsal* poses, research efforts focusing on this new pathogen have intensified, foremost in Europe. Studies have revealed which species are being affected by *Bsal* and how it is spreading (e.g., Spitzen-van der Sluijs et al. 2016; Lastra González et al. 2019). This research is complemented by monitoring studies in locations where *Bsal* is yet to be detected (e.g., Parrot et al. 2017; Waddle et al. 2020).

While data on *Bsal’s* host and invasion range are becoming increasingly available, it is dispersed in a multitude of publications. Pieces of information, such as the number of known *Bsal* hosts or the species in which *Bsal*-related deaths have been reported, are thus not always straightforward to obtain. This can cause problems, as researchers might not be aware of all available information, and decision-makers might not be able to react on time. For example, the first field report of *Bsal* detection in Spain (Lastra González et al. 2019) was neither cited nor discussed when the pathogen was re-discovered in Spain later on (Martel et al. 2020).

Now, almost ten years after *Bsal* was first reported, we believe it is useful to compile and review the available information. We therefore conducted a systematic review of published papers and compiled a database with records of *Bsal* tests performed on amphibians in the field, in captivity, or experimental setups. Similar efforts to compile records of infection have been undertaken in the past with the related pathogen *Bd* by Fisher et al. (2009), Castro Monzon et al. (2020) and Olson et al. (2014, 2021,2013). Such efforts have helped to better understand *Bd* infection. For *Bsal*, Baláž et al. (2017) published a systematic review focusing on Europe, comprising data up to 2016. That review gathered data from 30 publications and identified positive tests in 12 European amphibian species and three Asian ones. Since its publication, a large number of reports have appeared, for example in a special issue of the journal *Salamandra* in 2020 (e.g., Lötters et al. 2020a, b; Schulz et al. 2020). In our study, we included articles published up to 2021, and thus more than doubled the number of publications included in the last systematic *Bsal* review (Baláž et al. 2017).

Here, we aim to evaluate and condense the current understanding of *Bsal* host species and the pathogen’s invasive range. Specifically, we seek to understand which species have been reported with positive *Bsal* tests, in which species *Bsal*-related deaths have been reported, and to which families susceptible species belong to. We also seek to understand how many studies have been conducted in different countries, how often, and when the most recent study took place. Our literature review and summary provide insight into the threat that *Bsal* poses, and can inform management and research priorities.

## Methods

We conducted our work following the PRISMA guidelines (Preferred Reporting Items for Systematic Reviews and Meta-Analyses) as per Moher et al. (2010). We did this to increase repeatability and to ensure that this study and its accompanying dataset can be updated and extended as new data is published. For this purpose, we openly provide our dataset as supplementary material (Supplementary materials 1–6).

We searched the Web of Science for peer-reviewed articles on March 4, 2021, using the query: (chytrid* OR batrachochytrium) AND (amphibian* OR frog* OR salamander* OR anuran* OR urodelan* OR caudat* OR caecilian*). In addition, we also manually searched correspondence articles from the journal *Salamandra* up to the first volume of 2021. Similarly, we included articles from the journal *Herpetological Review* up to the first volume of 2021. We added publications from this non-indexed journal to our database, as it contains a section for papers related to amphibian diseases. In both *Herpetological Review* and *Salamandra,* we looked for articles containing the words “batrachochytrium” or “chytrid.”

Our query returned several articles where amphibians were tested for *Bd*, several of which also presented data on *Bsal.* We did not record data on *Bd* for this study, as that was recently done by Castro Monzon et al. (2020) and Olson et al (2021). We only selected articles where amphibian hosts were tested for *Bsal* either via qPCR or histological analysis. This included papers that reported tests in amphibians collected in the wild or kept in captivity, or in preserved amphibians from museums. Papers where amphibians were tested in an experimental setting, defined by the intentional exposure of amphibians to *Bsal*, were also included.

We included each relevant publication in our dataset and recorded the following data: names of the tested amphibians, the country where the amphibians were tested, the first and last year amphibians of a species was tested, the first and last year the species tested positive, the number of amphibians tested, how many *Bsal*-related deaths were reported and the coordinates of sampled locations. Species names as reported were checked against and updated (when necessary and possible) with species names in the Amphibian Species of the World Database (Frost 2021). Data from hybrids, kleptons and organisms with unresolved taxonomic status were recorded, but not included in the analysis.

## Results

*Bsal*-positive tests have been reported in a total of 67 species in ten different amphibian families. *Bsal*-related deaths have been reported in 29 species, all of them caudates from the Salamandridae and Plethodontidae families. Twenty-one of the species that have been reported with positive tests have a threat status (critically endangered, endangered or vulnerable) according to the IUCN Red List (IUCN 2021). *Bsal*-related deaths have been reported in eight of these threatened species (Table 1). Additionally, nine of the *Bsal*-positive species are known or suspected to be invasive (data from Frost 2021).

**Table 1:** All 67 Species that Have Tested Positive for *Bsal* in the Field, in Captivity or in Laboratory.

Distribution	Family	IUCN	Species	Field	Captivity	Experiment
AF	Salamandridae	**VU**	*Pleurodeles nebulosus*	NA	Positive	NA
AF/EU	Salamandridae	NT	*Pleurodeles waltl*	Negative	Positive	**Deaths**
AF/EU	Salamandridae	**VU**	*Salamandra algira*	Negative	**Deaths**	NA
EU	Alytidae	LC	Alytes obstetricans*	Negative	Negative	Positive
EU	Salamandridae	LC	*Lissotriton boscai*	Negative	Positive	NA
EU	Proteidae	**VU**	Proteus anguinus*	NA	Negative	Positive
EU	Salamandridae	**CR**	*Calotriton arnoldi*	Negative	NA	**Deaths**
EU	Salamandridae	**EN**	*Euproctus platycephalus*	Negative	Negative	**Deaths**
EU	Salamandridae	**VU**	*Lyciasalamandra helverseni*	Negative	NA	**Deaths**
EU	Salamandridae	LC	*Triturus marmoratus*	Positive	**Deaths**	**Deaths**
EU	Salamandridae	LC	*Salamandra salamandra*	**Deaths**	**Deaths**	**Deaths**
EU	Salamandridae	LC	*Lissotriton helveticus*	Positive	**Deaths**	Positive
EU	Salamandridae	LC	*Triturus cristatus*	Positive	**Deaths**	**Deaths**
EU	Salamandridae	LC	Ichthyosaura alpestris*	**Deaths**	Positive	**Deaths**
EU	Salamandridae	LC	*Lissotriton italicus*	Negative	Negative	**Deaths**
EU	Salamandridae	LC	*Salamandrina perspicillata*	NA	Negative	**Deaths**
EU	Plethodontidae	NT	Speleomantes strinatii*	Negative	Negative	**Deaths**
EU	Salamandridae	LC	*Salamandra atra*	Negative	Positive	NA
EU	Salamandridae	LC	*Salamandra corsica*	NA	Positive	NA
EU	Salamandridae	NT	*Triturus dobrogicus*	NA	Positive	NA
EU/CA/EA	Hynobiidae	LC	*Salamandrella keyserlingii*	Positive	Negative	Positive
EU/CA	Ranidae	LC	Rana temporaria*	Positive	NA	Negative
EU/CA	Salamandridae	LC	*Triturus karelinii*	NA	Positive	NA
EU/CA	Salamandridae	NT	Ommatotriton ophryticus*	Negative	Positive	NA
EU/CA	Salamandridae	NA	*Triturus ivanbureschi*	NA	Positive	NA
EU/CA	Salamandridae	LC	Lissotriton vulgaris*	Positive	Negative	**Deaths**
EU/CA	Salamandridae	NA	Triturus anatolicus*	Positive	Negative	Positive
CA	Salamandridae	NA	*Triturus macedonicus*	NA	**Deaths**	NA
CA	Salamandridae	**VU**	*Neurergus crocatus*	NA	Negative	**Deaths**
CA	Salamandridae	**VU**	*Neurergus strauchii*	NA	**Deaths**	NA
CA	Salamandridae	NT	*Salamandra infraimmaculata*	NA	Positive	NA
EA	Cryptobranchidae	**CR**	*Andrias davidianus*	NA	Positive	NA
EA	Salamandridae	**VU**	*Paramesotriton fuzhongensis*	Negative	Positive	NA
EA	Salamandridae	**VU**	*Tylototriton ziegleri*	Positive	negative	NA
EA	Bombinatoridae	**VU**	*Bombina microdeladigitora*	Positive	Positive	NA
EA	Salamandridae	**EN**	*Cynops ensicauda*	Positive	Positive	NA
EA	Salamandridae	**EN**	*Cynops orphicus*	Positive	NA	NA
EA	Salamandridae	**EN**	*Pachytriton wuguanfui*	Positive	NA	NA
EA	Salamandridae	**EN**	*Tylototriton vietnamensis*	Positive	Positive	NA
EA	Salamandridae	**VU**	*Paramesotriton aurantius*	Positive	NA	NA
EA	Salamandridae	**VU**	*Tylototriton wenxianensis*	Negative	Negative	**Deaths**
EA	Salamandridae	LC	*Cynops cyanurus*	Positive	Positive	**Deaths**
EA	Salamandridae	LC	*Cynops pyrrhogaster*	Positive	Negative	**Deaths**
EA	Salamandridae	LC	*Paramesotriton deloustali*	Positive	Positive	**Deaths**
EA	Salamandridae	NT	*Paramesotriton hongkongensis*	Positive	Positive	NA
EA	Salamandridae	NT	*Tylototriton asperrimus*	Positive	Negative	NA
EA	Salamandridae	NA	*Tylototriton uyenoi*	Positive	NA	NA
EA	Salamandridae	LC	*Tylototriton verrucosus*	Positive	Negative	NA
EA	Salamandridae	LC	*Cynops orientalis*	Positive	Negative	NA
EA	Hynobiidae	LC	*Hynobius nebulosus*	Positive	NA	NA
EA	Hynobiidae		*Hynobius sonani*	**Deaths**	NA	NA
AM	Ambystomatidae	LC	*Ambystoma opacum*	Negative	Positive	Negative
AM	Ambystomatidae	LC	*Ambystoma maculatum*	Negative	Positive	Positive
AM	Plethodontidae	**VU**	*Desmognathus apalachicolae*	NA	NA	Positive
AM	Salamandridae	**EN**	*Notophthalmus meridionalis*	NA	NA	**Deaths**
AM	Salamandridae	LC	*Notophthalmus viridescens*	Negative	**Deaths**	**Deaths**
AM	Salamandridae	LC	Taricha granulosa*	Negative	Negative	**Deaths**
AM	Plethodontidae	LC	*Desmognathus auriculatus*	Negative	NA	**Deaths**
AM	Plethodontidae	LC	*Eurycea cirrigera*	Negative	Negative	**Deaths**
AM	Plethodontidae	LC	*Eurycea wilderae*	Negative	NA	**Deaths**
AM	Plethodontidae	LC	*Pseudotriton ruber*	Negative	Negative	**Deaths**
AM	Plethodontidae	NT	*Aquiloeurycea cephalica*	NA	Negative	Positive
AM	Plethodontidae	LC	*Ensatina eschscholtzii*	Negative	Negative	Positive
AM	Plethodontidae	LC	*Eurycea guttolineata*	Negative	NA	Positive
AM	Plethodontidae	LC	*Eurycea lucifuga*	NA	NA	Positive
AM	Plethodontidae	NA	*Desmognathus conanti*	NA	NA	Positive
AM	Sirenidae	LC	*Siren intermedia*	Negative	Negative	Positive

Species are ordered by region of origin. Species distribution is marked in the following way *AF* North Africa, *EU* Europe, *CA* Central Asia, *EA* East and Southeast Asia, *AM* Americas. Suspected invasive species are marked with an asterisk. IUCN Red List status is indicated as follows: *LC* Least concern, *NT* Near threatened, *VU* Vulnerable, *EN* Endangered, *CR* Critically endangered; VU, EN and CR are highlighted in bold. *Bsal*-related deaths are highlighted in bold as well. When no data is available, NA is used (data in supplementary materials 1–3).

Our data comes from 65 papers that reported tests for *Bsal* in amphibians; 46 of these papers also reported tests for *Bd*, usually by means of duplex qPCR as per Blooi et al. (2013). Reports of negative tests abound, with 30 papers only reporting negative tests. Tests come from 33 countries, although 16 of these countries have only been studied once (Table 2).

**Table 2:** The Number of Caudate Species Countries Where Tests have been Conducted in the Field.

	# Cau species	% Threatened (%)	# Known hosts	# Field infected	# Field tested	# Studies
USA	210	23	15	0	94	18
Mexico	153	81	4	0	7	3
China	81	66	15	8	55	4
Guatemala	65	82	0	0	6	1
Japan	52	43	4	4	12	1
Panama	33	50	0	0	61	1
Canada	22	0	4	0	1	1
Turkey	21	60	8	0	8	2
Italy	19	37	10	0	13	2
France	14	15	11	0	3	2
Spain	12	27	11	4	16	5
Vietnam	9	50	4	6	40	4
Germany	9	0	8	6	11	13
Czechia	8	0	7	0	3	2
Croatia	8	14	7	0	2	2
Greece	8	28	6	0	1	1
Taiwan	7	83	2	1	1	1
Austria	7	0	6	0	2	2
Switzerland	7	0	8	0	7	2
Thailand	7	0	2	1	3	1
Netherlands	6	0	7	3	9	8
Slovakia	6	0	6	0	1	1
Montenegro	6	16	6	0	2	1
Belgium	5	0	7	2	7	5
Laos	5	40	0	0	1	1
Poland	5	0	5	0	1	1
UK	5	0	6	0	4	4
Peru	5	0	0	0	1	1
Morocco	2	50	2	0	1	1
Brunei	0	0	0	0	28	1
Malaysia	0	0	0	0	25	1

The number of caudate species in each country is shown (Frost 2021). Also presented is the percentage of caudate species with threatened conservation status (critically endangered, endangered or vulnerable). Additionally, the number of known *Bsal* susceptible amphibian species that exist in a country is presented as well as the number of species that have tested positive in the field, the number of species tested and the number of studies that took place in that country (references for studies in each country in supplementary materials 4).

**Figure 1:**
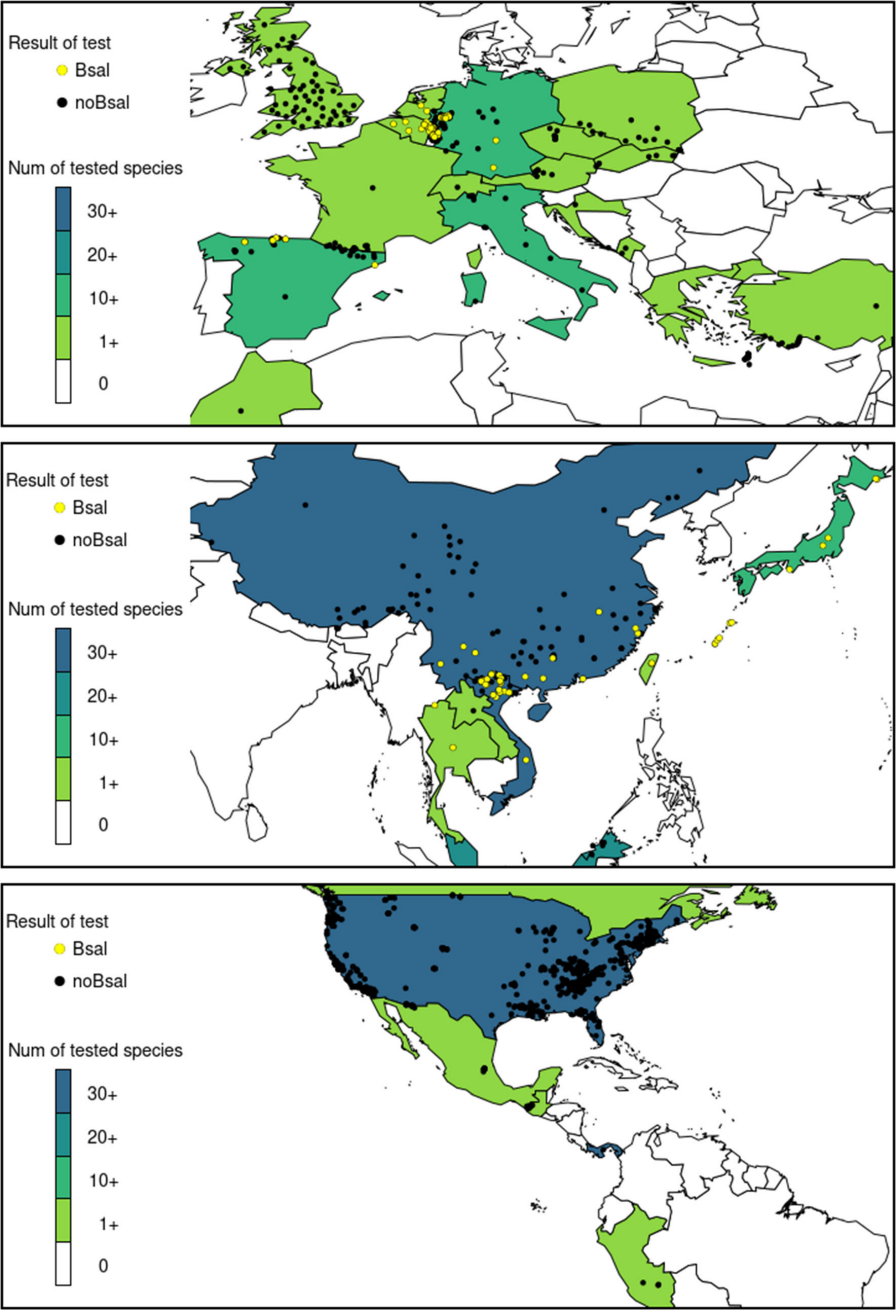
Number of amphibian species tested in each country in the field, the location and results found in sampled locations.

**Figure 2:**
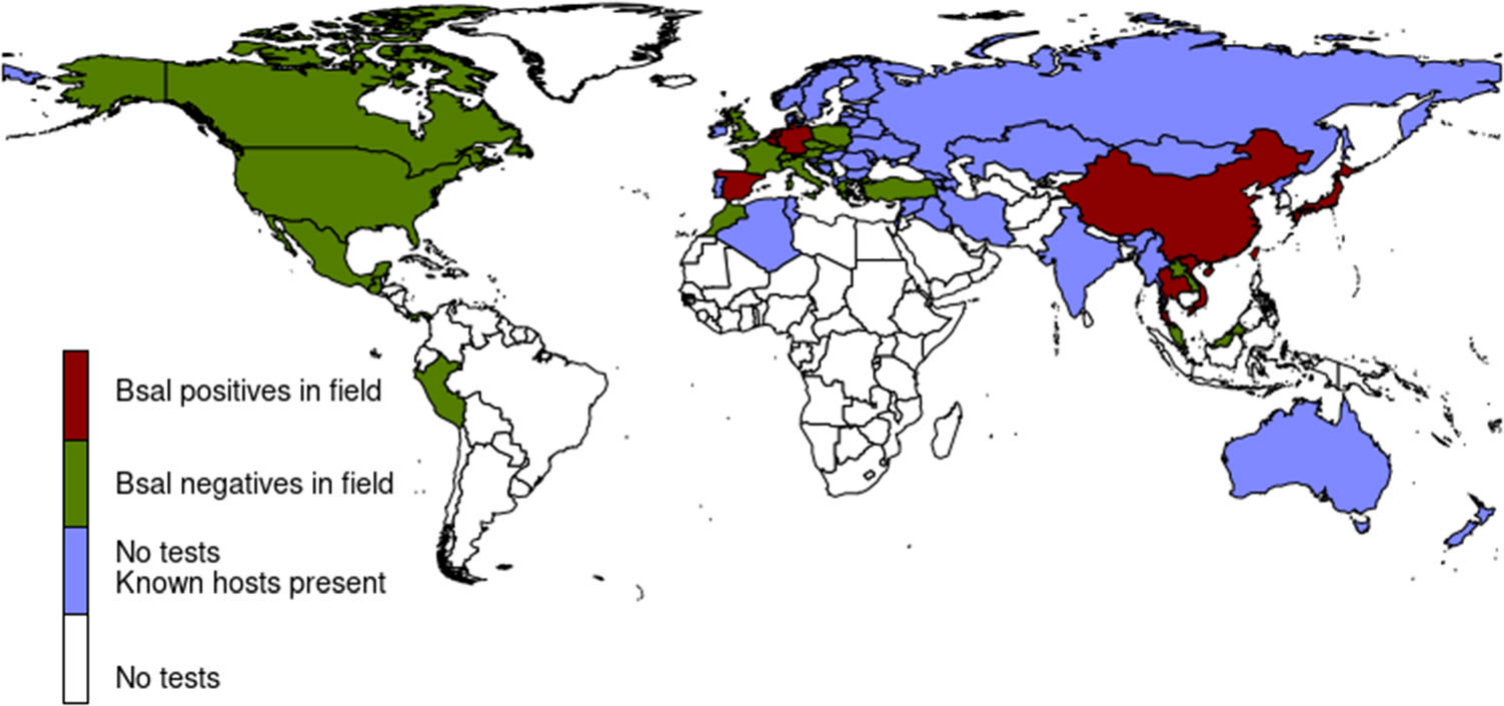
Countries in which *Bsal* has been found in the field, has not been found or where no tests have been conducted, but where known hosts are distributed. Notice that both the native and non-native distribution of host species is marked (e.g., *Lissotriton vulgaris* is found in Australia). Known *Bsal* host species exist in all colored countries except Peru, Venezuela, Lao, Brunei and Malaysia.

**Figure 3:**
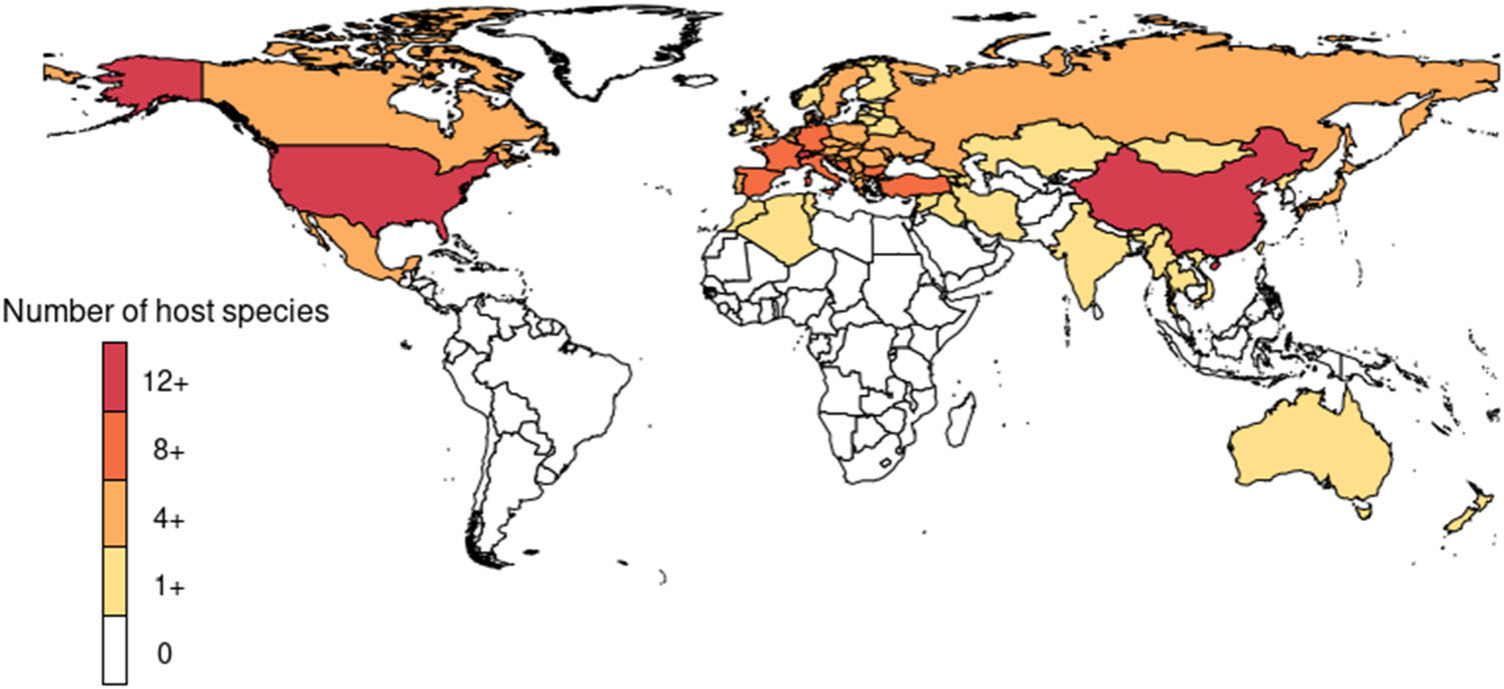
Countries are colored differently to highlight the number of known host species that are distributed there. Notice that both the native and nonnative distribution of host species is marked (e.g., *Lissotriton vulgaris* is found in Australia).

Not surprisingly, a large number of studies come from Germany, Belgium, and the Netherlands, countries in which *Bsal* was detected several years ago (*n* = 18). An equally large number of studies came from the United States (*n* = 18). Data from Mexico and Central America is scarce (*n* = 4) and even completely absent for some countries (Figs. 1, 2). We found reports of tests in the field from 31 countries, but positives have only been reported in nine (Table 2). The native and non-native distribution of species that are known *Bsal* hosts encompasses 69 countries (Fig. 3). These countries hold ~ 75% of all existing caudate species.

In Europe, *Bsal* has been tested in the field in 15 countries, but positives have so far only been found in the Netherlands, Belgium, Germany, and Spain (Figs. 1, 2). There are 26 European species which are known *Bsal* hosts (Table 1) but, in the field, the pathogen has only been detected in eight of them. *Bsal*-related deaths have been reported in 14 European amphibian species (Table 1), all but one (the plethodontid *Speleomantes strinatii*) are salamandrids. Important for conservation purposes is the report of *Bsal*-deaths in five threatened species; fortunately, infection in these species is yet to be found in the field (Table 1). Also relevant is the detection of *Bsal* in a museum specimen collected in Germany in 2004, that is the earliest known record of the pathogen in Europe (Lötters et al. 2020a).

Amphibian species from East Asia have been tested in eight countries: China, Taiwan, Japan, Thailand, Vietnam, Laos, Malaysia, and Brunei (Fig. 1). *Bsal* has been detected in 20 species from East and Southeast Asia (Table 1) and in the field in 19 of these species. One of the species that tested positive for *Bsal*, *Bombina microdeladigitora,* is an anuran, and there is a report of this frog testing positive both in the field and in captivity. Also testing positive were two salamander species of Hynobiidae family and one of Cryptobranchidae. The remaining infected species were all members of Salamandridae. There is evidence suggesting that some Asian amphibians might be susceptible as *Bsal*-related deaths have been reported in the field in *Hynobious sonani* (Beukema et al. 2018) and from laboratory exposure experiments in five species, two of which have a threatened conservation status (Table 1). Of interest is the detection of *Bsal* in a preserved *Cynops ensicauda* specimen dating from 1861 (Martel et al. 2014). At the time in which this manuscript is being written, this is the oldest known *Bsal* record. *Cynops ensicauda* is only distributed in Japan (Sparreboom 2014) and, presumably, the infected specimen originated there.

In the Americas, tests for *Bsal* have been conducted in the field in the USA, Canada, Mexico, Guatemala, Panama, and Peru (Fig. 1). *Bsal* is yet to be found in the Americas although laboratory exposure experiments and reports of infection in captivity show that at least 16 species native to the Americas are capable of becoming infected with *Bsal* (Table 1)*.* These include species from the Salamandridae, Ambystomatidae, Sirenidae, and Plethodontidae families*.* Most importantly, *Bsal*-related deaths have been reported in seven species, four of which were plethodontids.

The number of species that have been detected with *Bsal* has grown since the pathogen was discovered (Supplementary materials 7). This is mostly due to reports of species tested in the laboratory or in captivity. It reflects an accumulation of knowledge as new species are tested or as infection is tested in less susceptible species. The number of species found with *Bsal* in the field has also increased through time. Most of these species (19 out of 26) are distributed in East and Southeast Asia (Table 1).

## Discussion

The number of papers reporting *Bsal* tests had initially grown slowly. Less than five papers were published each year up to 2016, 10 papers each year thereafter, and 24 papers in 2020. The publication peak in 2020 is extraordinary when compared with previous years, although it is partially explained by the publication of a large number of papers in a *Bsal*-dedicated issue (vol. 56) of the journal *Salamandra*. Most of the papers we found that tested for *Bsal* in the wild or in captivity also tested for *Bd,* usually with the duplex method developed by Blooi et al. (2013)*.* The converse is not true, though: most of the papers that test for *Bd* do not conduct tests for *Bsal*. Lötters et al. (2020a, b) argued for the importance of histological analysis in identifying *Bsal,* but this method has not been widely adopted and has only been reported in studies from the Netherlands, Belgium, and Germany. Histological analysis is, undoubtedly, a useful tool (Lötters et al. 2020a, b) but its use is not widespread, likely because of practical reasons (e.g., scalability and level of expertise required).

The data gathered and analyzed in this study appears to support Martel et al.’s (2014) observation of a higher susceptibility for *Bsal* in caudate compared to anuran species*.* We found reports of positive tests in amphibians from seven caudate families. In contrast, reports of positive tests comprised only three anuran families. Moreover, most of the species that were reported with positive tests (64/67) were caudates. However, we urge caution as only 10 anuran species have been tested in exposure experiments. We found no reports of *Bsal*-related deaths among anurans in our data, but the detection of *Bsal* in *Bombina microdeladigitora* specimens, kept in a German pet shop, raises concerns about the possibility of anurans being a reservoir for the pathogen (Nguyen et al. 2017). Work that was published after our data collection (Towe et al. 2021) has shown that *Bsal* can infect a frog species from the Americas (*Osteopilus septentrionalis*) and, more so, that infection can result in chytridiomycosis which can be lethal. This should be a warning and call to action to investigate the potential effects that *Bsal* could have on non-caudate amphibians (anurans and caecilians).

In captivity, reports of infection appear more frequently in salamandrid than non-salamandrid caudates (even when accounting for the number of individuals tested). Infection in plethodontid caudates is also noteworthy; Plethodontidae is the only family, besides Salamandridae, in which *Bsal*-related deaths have been reported (Martel et al. 2014; Carter et al. 2019; Friday et al. 2020). This suggests that, while *Bsal* is able to infect a wide range of amphibian species, it affects predominantly salamandrid and plethodontid caudates.

The reports of *Bsal*-related deaths in 14 European salamander species show the potential risk that the pathogen poses. As of now, *Bsal* has been detected in the field in eight European species (Table 1). However, *Bsal*-related deaths in the field have only been reported in *Salamandra salamandra* and, more recently, in *Ichthyosaura alpestris* (Schmeller et al. 2020). Populations of *S. salamandra* have been reported to be affected or even to become locally extinct in regions of the Netherlands, Belgium, and Germany (Martel et al. 2014; Schulz et al. 2020). A potential threat for other species is the expanding range of *Bsal* (Spitzen-van der Sluijs et al. 2016) or the translocation of infected individuals into a new area. Species with threatened conservation status and small distribution areas are particularly susceptible (see for example Martel et al. 2020). There are 12 European threatened caudate species; all but three have been tested in the field, the exceptions being: *Chioglossa lusitanica*, *Proteus anguinus,* and *S. lanzai*. In the field, tested threatened European species only returned negatives. However, tests from individuals kept in captivity or exposed show that at least five of these threatened species can carry infection. These species are: *Calotriton arnoldi*, *Euproctus platycephalus*, *Lyciasalamandra helverseni*, *S. algira,* and *P. anguinus*. Further, *Bsal*-related deaths have been reported in all these species except *P. anguinus* (Sabino-Pinto et al. 2015; Martel et al. 2014, 2020; Li et al. 2020)*.* The recognition of a taxonomic susceptibility in plethodontid caudates also highlights the risk that European cave salamanders (*Speleomantes* sp*.*) face.

As mentioned before, the origin of *Bsal* has been postulated to be in East Asia (Martel et al. 2014). The species that evolved with the pathogen presumably have a greater resistance to infection. However, the historic distribution of *Bsal* is not known, and some species in Asia might be susceptible. Interestingly, Martel et al. (2014) report *Bsal*-related deaths from exposure experiments in four East Asian caudate species: *Cynops cyanurus*, *C. pyrrhogaster*, *Paramesotriton deloustali,* and *Tylototriton wenxianensis* (Table 1)*.* These species have distributions that include Japan, central and southern China, and north Vietnam (Frost 2021; IUCN 2021). There are 89 threatened caudate species in East Asia and South East Asia, and only 40 have been tested, 25 of them in the field. We thus still know little about the susceptibility of Asian species to *Bsal*.

The Americas are incredibly important for caudate diversity, as 538 species of the 760 known caudate species live in this region (Frost 2021). *Bsal* has not been detected in the field in the Americas, nor has it been found in captive animals there. However, most of the species in the Americas belong to the Plethodontidae family which, as previously discussed, has shown to be susceptible in exposure experiments. We found reports of infection in laboratory exposures from amphibians kept in captivity from 16 species distributed in the Americas (Table 1) and reports of *Bsal*-related deaths in seven of them (Table 1). These species are distributed in the USA, Canada, and Mexico. Species distributed in Central America have not been tested in laboratory exposure experiments, which unfortunately leaves us with little information to evaluate the susceptibility of species from that region.

In the field, there have been 15 prospective studies in the USA, while only three were conducted in Mexico (Ellison et al. 2019; Olivares Miranda et al. 2020; Waddle et al. 2020) and two in Central America (Table 2). This difference is expected; research within each country is affected by economic, social and political circumstances, and spatial research biases are known for many other topics (e.g., Tydecks et al. 2018; Jeschke et al. 2019). This is nonetheless a critical problem, as the small number of prospective studies in a region might not allow for timely detection of infection. Pathogens and their hosts are also not bound by political borders. Hence, the establishment of infection in a region places species in neighboring countries at greater risk. Mexico and Central America hold more than half of the known caudate species, 74% of which are being threatened and models suggest that there are areas suitable for *Bsal* in these countries (Basanta et al. 2019, García-Rodríguez 2022).

Special attention should be taken to invasive amphibian species, potentially acting as vectors and reservoirs of the pathogen. Invasive caudate species might not be as infamous as their anuran counterparts (e.g., *Rhinella marina*, *Lithobates catesbeianus, Xenopus laevis*); however, infected caudates might bring the pathogen with them and can be transported long distances (Fisher & Garner 2007; Falaschi et al. 2020). For example, one of the species found with *Bsal*, *Lissotriton vulgaris*, has often been translocated in Europe and has even established a population in Australia (Tingley et al. 2015; Dubey et al. 2018). Another species found with *Bsal*, *I. alpestris,* has established populations in the UK, New Zealand, southern France, and Spain (Bell 2016; Frost 2021). Martel et al. (2020) argued that the presence of introduced species such as *I. alpestris* and *Triturus anatolicus* in Spain is associated with the introduction of *Bsal* there.

## Conclusions

The data presented in this study should help to plan future *Bsal* studies and protect vulnerable species. The 67 species in the list of known *Bsal* hosts should be carefully studied, and considerations should be taken to evaluate the risk that *Bsal* poses to these species. *Bsal*-related deaths have been reported in several threatened host species. This suggests a susceptibility in species that already face conservation challenges. Threatened species in Europe that are susceptible, such as *Calotriton arnoldi, Euproctus platycephalus, Lyciasalamandra helverseni,* and *Salamandra algira,* might be particularly vulnerable, as the pathogen has been found both in the field and in captivity within their native range. Areas where threatened and susceptible European species are distributed should be constantly monitored. We assume that some species in Asia evolved with the pathogen and might be resistant, but the native range of *Bsal* is still unclear, and we found at least one report of *Bsal*-related deaths in Asian threatened species.

The data compiled here may also help to establish, evaluate and update measures to prevent the arrival of the pathogen in areas where it has not yet been detected. The USA, for example, implemented measures to restrict the import of a large number of amphibians on a list (50 CFR § 16.14). However, that list does not currently include 12 amphibian species of which we found infection reports. Of these species, six have been reported or are suspected to have colonized areas outside their native range in the past (Table 1). This furthers our understanding of species that pose a risk as *Bsal* carriers. In at least one case, *Bsal* spread has been associated with the presence of an invasive species (Martel et al. 2020). The list of infected species provided in this work may serve as a guiding tool for decision makers on which species are at risk.

Tests involving experimental exposure previously showed that some salamandrid and plethodonthid species are susceptible. In this study, we showed that this susceptibility extends to a large number of species in those families, strongly suggesting the existence of a taxonomic pattern of susceptibility. The apparent existence of susceptibility in Plethodonthidae highlights the risk that *Bsal* poses in North and Central America. Our study also highlights countries and amphibians from specific regions that need prospective studies, namely Mexico and Central America. Constant monitoring is key, as timely detection of infection is of utmost importance to protect vulnerable species. Extirpation of the pathogen becomes harder, if not impossible, once the pathogen has spread to a large area.

## Supplementary Information

Below is the link to the electronic supplementary material.

Supplementary file1 (ODT 22 kb)

Supplementary file2 (ODT 32 kb)

Supplementary file3 (CSV 226 kb)

Supplementary file4 (CSV 2 kb)

Supplementary file5 (CSV 2 kb)

Supplementary file6 (CSV 119 kb)

Supplementary file7 (TIFF 1835 kb)
