# Supplementary material for: Batrachochytrium salamandrivorans’ Amphibian Host Species and Invasion Range
Source: Ecohealth. 2023 Jan 7;19(4):475–86. doi: 10.1007/s10393-022-01620-9 (PMC9898388; doi:10.1007/s10393-022-01620-9)
Supplement: Supplementary file 2 [file 10393_2022_1620_MOESM2_ESM.odt]

Bibliography referenced in other tables

Balaz V, Solsky M, Gonzalez DL, Havlikova B, Zamorano JG, Sevilleja CG, Torrent L, Vojar J (2018) First survey of the pathogenic fungus Batrachochytrium salamandrivorans in wild and captive amphibians in the Czech Republic. Salamandra 54:87–91

Bales EK, Hyman OJ, Loudon AH, Harris RN, Lipps G, Chapman E, Roblee K, Kleopfer JD, Terrell KA (2015) Pathogenic chytrid fungus Batrachochytrium dendrobatidis, but not B. salamandrivorans, detected on eastern hellbenders. PLOS ONE 10:e0116405; DOI: 10.1371/journal.pone.0116405

Barnhart K, Bletz MC, Labumbard B, Tokash-Peters A, Gabor C, Woodhams DC (2020) Batrachochytrium salamandrivorans elicits acute stress response in spotted salamanders but not infection or mortality. Animal Conservation 23:533–546; DOI: 10.1111/acv.12565

Bates KA, Shelton JMG, Mercier VL, Hopkins KP, Harrison XA, Petrovan SO, Fisher MC (2019) Captivity and infection by the fungal pathogen Batrachochytrium salamandrivorans perturb the amphibian skin microbiome. Frontiers in Microbiology 10:1834; DOI: 10.3389/fmicb.2019.01834

Beukema W, Martel A, Nguyen TT, Goka K, Schmeller DS, Yuan ZY, Laking AE, Nguyen TQ, Lin CF, Shelton J, Loyau A, Pasmans F (2018) Environmental context and differences between native and invasive observed niches of Batrachochytrium salamandrivorans affect invasion risk assessments in the Western Palaearctic. Diversity and Distributions 24:1788–1801; DOI: 10.1111/ddi.12795

Beukema W, Pasmans F, Van Praet S, Ferri-Yanez F, Kelly M, Laking AE, Erens J, Speybroeck J, Verheyen K, Lens L, Martel A (2021) Microclimate limits thermal behaviour favourable to disease control in a nocturnal amphibian. Ecology Letters 24:27–37; DOI: 10.1111/ele.13616

Blooi M, Pasmans F, Longcore JE, Spitzen-van der Sluijs A, Vercammen F, Martel A (2013) Duplex Real-Time PCR for rapid simultaneous detection of Batrachochytrium dendrobatidis and Batrachochytrium salamandrivorans in amphibian samples. Journal of Clinical Microbiology 51:4173–4177; DOI: 10.1128/JCM.02313–13

Blooi M, Pasmans F, Rouffaer L, Haesebrouck F, Vercammen F, Martel A (2015) Successful treatment of Batrachochytrium salamandrivorans infections in salamanders requires synergy between voriconazole, polymyxin E and temperature. Scientific Reports 5:11788; DOI: 10.1038/srep11788

Carter ED, Miller DL, Peterson AC, Sutton WB, Cusaac JPW, Spatz JA, Rollins-Smith LA, Reinert LK, Bohanon M, Williams LA, Upchurch A, Gray MJ (2020) Conservation risk of Batrachochytrium salamandrivorans to endemic lungless salamanders. Conservation Letters 13:e12675; DOI: 10.1111/conl.12675

Cunningham AA, Smith F, McKinley TJ, Perkins M, Fitzpatrick LD, Wright ON, Lawson B (2019) Apparent absence of Batrachochytrium salamandrivorans in wild urodeles in the United Kingdom. Scientific Reports 9:2831; DOI: 10.1038/s41598–019–39338–4

Ellison S, Rovito S, Parra-Olea G, Vasquez-Almazan C, Flechas SV, Bi K, Vredenburg VT (2019) The Influence of habitat and phylogeny on the skin microbiome of amphibians in Guatemala and Mexico. Microbial Ecology 78:257–267; DOI: 10.1007/s00248–018–1288–8

Fitzpatrick LD, Pasmans F, Martel A, Cunningham AA (2018) Epidemiological tracing of Batrachochytrium salamandrivorans identifies widespread infection and associated mortalities in private amphibian collections. Scientific Reports 8:13845; DOI: 10.1038/s41598–018–31800–z

Friday B, Holzheuser C, Lips KR, Longo AV (2020) Preparing for invasion: Assessing risk of infection by chytrid fungi in southeastern plethodontid salamanders. Journal of Experimental Zoology Part A-Ecological and Integrative Physiology 33:829–840; DOI: 10.1002/jez.2427

Gimeno A, Meikl M, Pitt A, Winkler M, Berninger UG (2015) Testing of fire salamanders around Salzburg for Batrachochytrium salamandrivorans within a school project. Journal on Protected Mountain Areas Research and Management 7:72–76; DOI: 10.1553/eco.mont-7–1s72

Glorioso BM, Waddle JH, Richards-Zawacki CL (2017) Prevalence of Batrachochytrium dendrobatidis and B. salamandrivorans in the Gulf Coast Waterdog, Necturus beyeri, from Southeast Louisiana, USA. Herpetological Review 48:360–363

Gonzalez DL, Balaz V, Solsky M, Thumsova B, Kolenda K, Najbar A, Najbar B, Kautman M, Chajma P, Balogova M, Vojar J (2019) Recent findings of potentially lethal salamander fungus Batrachochytrium salamandrivorans. Emerging Infectious Diseases 25:1416–1418; DOI: 10.3201/eid2507.181001

Govindarajulu P, Matthews E, Ovaska K (2017) Swabbing for Batrachochytrium salamandrivorans on wild Rough-Skinned Newts (Taricha granulosa) and pet-traded amphibians on Southern Vancouver Island, British Columbia, Canada. Herpetological Review 48:564–568

Grasselli E, Bianchi G, Dondero L, Marchiano V, Carafa M, Perrone M, Salvidio S (2019) First screening for Batrachochytrium salamandrivorans (Bsal) in wild and captive salamanders from Italy. Salamandra 55:124–126

Greener MS, Verbrugghe E, Kelly M, Blooi M, Beukema W, Canessa S, Carranza S, Croubels S, De Troyer N, Fernandez-Giberteau D, Goethals P, Lens L, Li ZM, Stegen G, Strubbe D, van Leeuwenberg R, Van Praet S, Vila-Escale M, Vervaeke M, Pasmans F, Martel A (2020) Presence of low virulence chytrid fungi could protect European amphibians from more deadly strains. Nature Communications 11:5393; DOI: 10.1038/s41467–020–19241–7

Guthrie A, Guthrie R, Steele K (2017) Batrachochytrium dendrobatidis and Batrachochytrium salamandrivorans surveillance in salamanders of southeastern Virginia, USA. Herpetological Review 48:363–365

Hardman RH, Sutton WB, Irwin KJ, McGinnity D, Reinsch SD, Freake M, Colclough P, Miller BT, Neto JGD, Souza M, Fitzpatrick B, Miller DL (2020) Geographic and individual determinants of important amphibian pathogens in hellbenders (Cryptobranchus alleganiensis) in Tennessee and Arkansas, USA. Journal of Wildlife Diseases 56:803–814; DOI: 10.7589/2019–08–203

Horan S, McDonald C, Bletz MC (2020) Batrachochytrium dendrobatidis is widespread and associated with water temperature in the Eastern United States. Herpetological Review 51:484–488

Ip HS, Lorch JM, Blehert DS (2016) Detection of spring viraemia of carp virus in imported amphibians reveals an unanticipated foreign animal disease threat. Emerging Microbes & Infections 5:e97; DOI: 10.1038/emi.2016.94

Iwanowicz DD, Schill WB, Olson DH, Adams MJ, Densmore C, Cornman RS, Adams C, Densmore C, Cornman RS, Adams C, Figiel Jr. C, Anderson CW, Blaustein AR, Chestnut T (2017) Potential concerns with analytical methods used for the detection of Batrachochytrium salamandrivorans from archived DNA of amphibian swab samples, Oregon, USA. Herpetological Review 48:352–355

Jung L, Dusek J, Luddecke T, Schulz V, Maier-Sam K, Habich L, Mosebach A, Lierz M, Ziemek HP (2020) Epidemiological screening of captive salamanders reveals current absence of Batrachochytrium salamandrivorans in private collections throughout the federal state of Hesse (Germany). Salamandra 56:233–238

Klocke B, Becker M, Lewis J, Fleischer RC, Muletz-Wolz CR, Rockwood L, Aguirre AA, Gratwicke B (2017) Batrachochytrium salamandrivorans not detected in US survey of pet salamanders. Scientific Reports 7:13132; DOI: 10.1038/s41598–017–13500–2

Kumar R, Malagon DA, Carter ED, Miller DL, Bohanon ML, Cusaac JPW, Peterson AC, Gray MJ (2020) Experimental methodologies can affect pathogenicity of Batrachochytrium salamandrivorans infections. PLOS ONE 15:9; DOI: 10.1371/journal.pone.0235370

Laking AE, Ngo HN, Pasmans F, Martel A, Nguyen TT (2017) Batrachochytrium salamandrivorans is the predominant chytrid fungus in Vietnamese salamanders. Scientific Reports 7:44443; DOI: 10.1038/srep44443

Le Sage EH, LaBumbard BC, Reinert LK, Miller BT, Richards-Zawacki CL, Woodhams DC, Rollins-Smith LA (2021) Preparatory immunity: Seasonality of mucosal skin defences and Batrachochytrium infections in southern leopard frogs. Journal of Animal Ecology 90:542–554; DOI: 10.1111/1365–2656.13386

Li ZM, Martel A, Bogaerts S, Gocmen B, Pafilis P, Lymberakis P, Woeltjes T, Veith M, Pasmans F (2020) Landscape connectivity limits the predicted impact of fungal pathogen invasion. Journal of Fungi 6:4; DOI: 10.3390/jof6040205

Li ZM, Verbrugghe E, Konstanjsek R, Lukac M, Pasmans F, Cizelj I, Martel A (2020) Dampened virulence and limited proliferation of Batrachochytrium salamandrivorans during subclinical infection of the troglobiont olm (Proteus anguinus). Scientific Reports 10:16480; DOI: 10.1038/s41598–020–73800–y

Longo AV, Fleischer RC, Lips KR (2019) Double trouble: co-infections of chytrid fungi will severely impact widely distributed newts. Biological Invasions 21:2233–2245; DOI: 10.1007/s10530–019–01973–3

Lotters S, Wagner N, Albaladejo G, Boning P, Dalbeck L, Dussel H, Feldmeier S, Guschal M, Kirst K, Ohlhoff D, Preissler K, Reinhardt T, Schlupmann M, Schulte U, Schulz V, Steinfartz S, Twietmeyer S, Veith M, Vences M, Wegge J (2020a) The amphibian pathogen Batrachochytrium salamandrivorans in the hotspot of its European invasive range: past - present – future. Salamandra 56:173–188

Lotters S, Veith M, Wagner N, Martel A, Pasmans F (2020b) Bsal-driven salamander mortality pre-dates the European index outbreak. Salamandra 56:239–242

Malagon DA, Melara LA, Prosper OF, Lenhart S, Carter ED, Fordyce JA, Peterson AC, Miller DL, Gray MJ (2020) Host density and habitat structure influence host contact rates and Batrachochytrium salamandrivorans transmission. Scientific Reports 10:5584; DOI: 10.1038/s41598–020–62351–x

Martel A, Spitzen-van der Sluijs A, Blooi M, Bert W, Ducatelle R, Fisher MC, Woeltjes A, Bosman W, Chiers K, Bossuyt F, Pasmans F (2013) Batrachochytrium salamandrivorans sp. nov. causes lethal chytridiomycosis in amphibians. Proceedings of the National Academy of Sciences of the United States of America 110:15325–15329; DOI: 10.1073/pnas.1307356110

Martel A, Blooi M, Adriaensen C, Van Rooij P, Beukema W, Fisher MC, Farrer RA, Schmidt BR, Tobler U, Goka K, Lips KR, Muletz C, Zamudio KR, Bosch J, Lotters S, Wombwell E, Garner TWJ, Cunningham AA, Spitzen-van der Sluijs A, Salvidio S, Ducatelle R, Nishikawa K, Nguyen TT, Kolby JE, Van Bocxlaer I, Bossuyt F, Pasmans F (2014) Recent introduction of a chytrid fungus endangers Western Palearctic salamanders. Science 346:630–631; DOI: 10.1126/science.1258268

Martel A, Vila-Escale M, Fernandez-Giberteau D, Martinez-Silvestre A, Canessa S, Van Praet S, Pannon P, Chiers K, Ferran A, Kelly M, Picart M, Piulats D, Li ZM, Pagone V, Perez-Sorribes L, Molina C, Tarrago-Guarro A, Velarde-Nieto R, Carbonell F, Obon E, Martinez-Martinez D, Guinart D, Casanovas R, Carranza S, Pasmans F (2020) Integral chain management of wildlife diseases. Conservation Letters 13:e12707; DOI: 10.1111/conl.12707

Martínez-Silvestre Albert, Trochet Audrey, Calvez Olivier, Poignet Manon, Le Chevalier Hugo, Souchet Jérémie, Darnet Elodie, Guillaume Olivier, Aubret Fabien, Bertrand Romain, Mossoll-Torres Marc, Lucati Federica, Tomàs Jan, O’Brien David, Miró Alexandre , Ventura Marc , Barthe Laurent, Pottier Gilles, Marschang Rachel E., Bosch Jaime (2020) Presence of the fungus Batrachochytrium dendrobatidis, but not Batrachochytrium salamandrivorans, in wild pyrenean brook newts (Calotriton asper) in Spain and France. Herpetological Review 51:738–743

McLeod DS, Gumpf J, Alavi B, Grafe TU, Yambun P, Hyman OJ (2020) Detection of Batrachochytrium dendrobatidis but not B. salamandrivorans in frog populations from Northern Borneo. Herpetological Review 51:257–261

Michaels CJ, Rendle M, Gibault C, Lopez J, Garcia G, Perkins MW, Camerons S, Tapley B (2018) Batrachochytrium dendrobatidis infection and treatment in the salamanders Ambystoma andersoni, A. dumerilii and A. mexicanum. Herpetological Journal 28:87–92

Muletz C, Caruso NM, Fleischer RC, McDiarmid RW, Lips KR (2014) Unexpected rarity of the pathogen Batrachochytrium dendrobatidis in appalachian plethodon salamanders. 1957–2011. PLOS ONE 9:e103728; DOI: 10.1371/journal.pone.0103728

Newman JC, Mota JL, Hardman RH, Dillman JW, Barrett K (2019) Pathogen detection in green salamanders (Aneides aeneus) in South Carolina, USA. Herpetological Review 50:503–505

Nguyen TT, Nguyen TV, Ziegler T, Pasmans F, Martel A (2017) Trade in wild anurans vectors the urodelan pathogen Batrachochytrium salamandrivorans into Europe. Amphibia-Reptilia 38:554–556; DOI: 10.1163/15685381–00003125

Olivares-Miranda, M, Vredenburg, VT, Garcia-Sanchez, JC, Byrne, AQ, Rosenblum, EB, Rovito, SM (2020) Fungal infection, decline and persistence in the only obligate troglodytic Neotropical salamander. PEERJ 8:e9763; DOI: 10.7717/peerj.9763

Ossiboff RJ, Towe AE, Brown MA, Longo AV, Lips KR, Miller DL, Carter ED, Gray MJ, Frasca S (2019) Differentiating Batrachochytrium dendrobatidis and B. salamandrivorans in amphibian chytridiomycosis using RNAScope (R) in situ hybridization. Frontiers in Veterinary Science 6:304; DOI: 10.3389/fvets.2019.00304

Parrott JC, Shepack A, Burkart D, LaBumbard B, Scime P, Baruch E, Catenazzi A (2017) Survey of pathogenic chytrid fungi (Batrachochytrium dendrobatidis and B-salamandrivorans) in salamanders from three mountain ranges in Europe and the Americas. Ecohealth 15:296–302; DOI: 10.1007/s10393–016–1188–7

Sabino-Pinto J, Bletz M, Hendrix R, Perl RGB, Martel A, Pasmans F, Lotters S, Mutschmann F, Schmeller DS, Schmidt BR, Veith M, Wagner N, Vences M, Steinfartz S. (2015) First detection of the emerging fungal pathogen Batrachochytrium salamandrivorans in Germany. Amphibia-Reptilia 36:411–416; DOI: 10.1163/15685381–00003008

Sabino-Pinto J Veith M Vences M Steinfartz S (2018) Asymptomatic infection of the fungal pathogen Batrachochytrium salamandrivorans in captivity. Scientific Reports 8:11767; DOI: 10.1038/s41598–018–30240–z

Sachs M, Schluckebier R, Poll K, Schulz V, Sabino-Pinto J, Schmidt E, Simon K, Kunzel S, Ziegler T, Arndt H, Vences M (2020) Evidence of Batrachochytrium dendrobatidis and other amphibian parasites in the Green toad (Bufotes viridis), syntopic amphibians and environment in the Cologne Bay, Germany. Salamandra 56:275–284

Sandvoss M, Wagner N, Lotters S, Feldmeier S, Schulz V, Steinfartz S, Veith M (2020) Spread of the pathogen Batrachochytrium salamandrivorans and large-scale absence of larvae suggest unnoticed declines of the European fire salamander in the southern Eifel Mountains. Salamandra 56:215–226

Schmeller DS, Utzel R, Pasmans F, Martel A (2020) Batrachochytrium salamandrivorans kills alpine newts (Ichthyosaura alpestris) in southernmost Germany. Salamandra 56:230–232

Schulz, V, Schulz, A, Klamke, M, Preissler, K, Sabino-Pinto, J, Musken, M, Schlupmann, M, Heldt, L, Kamprad, F, Enss, J, Schweinsberg, M, Virgo, J, Rau, H, Veith, M, Lotters, S, Wagner, N, Steinfartz, S, Vences, M (2020) Batrachochytrium salamandrivorans in the Ruhr District, Germany: history, distribution, decline dynamics and disease symptoms of the salamander plague. Salamandra 56:189–214

Spitzen-van der Sluijs A, Martel A, Asselberghs J, Bales EK, Beukema W, Bletz MC, Dalbeck L, Goverse E, Kerres A, Kinet T, Kirst K, Laudelout A, da Fonte LFM, Nollert A, Ohlhoff D, Sabino-Pinto J, Schmidt BR, Speybroeck J, Spikmans F, Steinfartz S, Veith M, Vences M, Wagner N, Pasmans F, Lotters S. (2016) Expanding distribution of lethal amphibian fungus Batrachochytrium salamandrivorans in Europe. Emerging Infectious Diseases 22:1286–1288; DOI: 10.3201/eid2207.160109

Spitzen-van der Sluijs A, Stegen G, Bogaerts S, Canessa S, Steinfartz S, Janssen N, Bosman W, Pasmans F, Martel A (2018) Post-epizootic salamander persistence in a disease-free refugium suggests poor dispersal ability of Batrachochytrium salamandrivorans. Scientific Reports 8:3800; DOI: 10.1038/s41598–018–22225–9

Standish I, Leis E, Schmitz N, Credico J, Erickson S, Bailey J, Kerby J, Phillips K, Lewis T (2018) Optimizing, validating, and field testing a multiplex qPCR for the detection of amphibian pathogens. Diseases of Aquatic Organisms 129:1–13; DOI: 10.3354/dao03230

Stegen G, Pasmans F, Schmidt BR, Rouffaer LO, Van Praet S, Schaub M, Canessa S, Laudelout A, Kinet T, Adriaensen C, Haesebrouck F, Bert W, Bossuyt F, Martel A (2017) Drivers of salamander extirpation mediated by Batrachochytrium salamandrivorans. Drivers of salamander extirpation mediated by Batrachochytrium salamandrivorans. Nature 544:353; DOI: 10.1038/nature22059

Tapley B, Cutajar T, Nguyen LT, Rowley JJL (2020) Low Prevalence of Batrachochytrium dendrobatidis detected in amphibians from Vietnam’s highest mountains. Herpetological Review 51:726–732

Vieira WA, Kelsey A, Campbell GL, McCusker CD (2020) Characterizing the regenerative capacity and growth patterns of the Texas blind salamander (Eurycea rathbuni). Developmental Dynamics 250:880–895; DOI: 10.1002/dvdy.245

Waddle JH, Grear DA, Mosher BA, Grant EHC, Adams MJ, Backlin AR, Barichivich WJ, Brand AB, Bucciarelli GM, Calhoun DL, Chestnut T, Davenport JM, Dietrich AE, Fisher RN, Glorioso BM, Halstead BJ, Hayes MP, Honeycutt RK, Hossack BR, Kleeman PM, Lemos-Espinal JA, Lorch JM, McCreary B, Muths E, Pearl CA, Richgels KLD, Robinson CW, Roth MF, Rowe JC, Sadinski W, Sigafus BH, Stasiak I, Sweet S, Walls SC, Watkins-Colwell GJ, White CL, Williams LA, Winzeler ME (2020) Batrachochytrium salamandrivorans (Bsal) not detected in an intensive survey of wild North American amphibians. Scientific Reports 10:13012; DOI: 10.1038/s41598–020–69486–x

Wang SP, Zhu W, Fan LQ, Li JQ, Li YM (2017) Amphibians testing negative for Batrachochytrium dendrobatidis and Batrachochytrium salamandrivorans on the Qinghai-Tibetan Plateau, China. Asian Herpetological Research 8:190–198; DOI: 10.16373/j.cnki.ahr.170010

Wilber MQ, Carter ED, Gray MJ, Briggs CJ (2021) Putative resistance and tolerance mechanisms have little impact on disease progression for an emerging salamander pathogen. Functional Ecology 35:847–859; DOI: 10.1111/1365–2435.13754

Yuan ZY, Martel A, Wu J, Van Praet S, Canessa S, Pasmans F (2018) Widespread occurrence of an emerging fungal pathogen in heavily traded Chinese urodelan species. Conservation Letters 11:e12436; DOI: 10.1111/conl.12436

Zhu W, Xu F, Bai CM, Liu X, Wang SP, Gao X, Yan SF, Li XP, Liu ZT, Li YM (2014) A survey for Batrachochytrium salamandrivorans in Chinese amphibians. Current Zoology 60:729–735; DOI: 10.1093/czoolo/60.6.729
